# Severe Household Food Insecurity and Malnutrition Among School‐Going Adolescents in South Punjab, Pakistan: Implications for Sustainable Development Goal 2 (A Cross‐Sectional Study)

**DOI:** 10.1002/puh2.70393

**Published:** 2026-09-29

**Authors:** Rameesha Shauket, Inibehe Ime Okon, Saira Maroof

**Affiliations:** ^1^ Registered Dietitian Nutritionist—Armed Forces Post Graduate Medical Institute National University of Medical Sciences Rawalpindi Pakistan; ^2^ Department of Research Medical Research Circle (MedReC) Bukavu D. R. Congo; ^3^ Quetta Institute of Medical Sciences National University of Medical Sciences Quetta Pakistan

**Keywords:** adolescents, food insecurity, malnutrition, overweight, Pakistan, stunting, Sustainable Development Goals, thinness

## Abstract

**Introduction:**

Food insecurity and malnutrition remain major public health concerns among adolescents in low‐ and middle‐income countries. Adolescence is a critical period for growth and development, yet school‐going adolescents remain underrepresented in nutrition research and policy in Pakistan. This study aimed to assess household food insecurity, nutritional status, and their association among school‐going adolescents in peri‐urban South Punjab.

**Methods:**

This cross‐sectional study included 384 school‐going adolescents aged 15–18 years from public and private schools in peri‐urban Lodhran district, South Punjab, Pakistan. Household food access was assessed using the Household Food Insecurity Access Scale (HFIAS), and nutritional status was classified using WHO BMI‐for‐age (BAZ) and height‐for‐age (HAZ) *z*‐scores. Data were collected using a pretested, interviewer‐administered questionnaire. Associations were examined using chi‐square and Fisher's exact tests, multivariable logistic regression (adjusted for age, sex, school type, family size, socioeconomic score, and school grade) for the continuous HFIAS score, and Benjamini–Hochberg correction for multiple comparisons.

**Results:**

The mean age was 16.6 ± 1.1 years, and 207 (53.9%) were female. Overall, 365 households (95.1%, 95% CI 92.4%–96.8%) were severely food insecure. Thinness was observed in 131 adolescents (34.1%, 95% CI 29.6%–39.0%), stunting in 107 (27.9%, 95% CI 23.6%–32.6%), and overweight/obesity in 35 (9.1%, 95% CI 6.6%–12.4%). Overall HFIAS categories were not significantly associated with stunting, thinness, or overweight/obesity. After correction for multiple comparisons, four individual HFIAS experience domains remained robustly associated with thinness specifically, and in multivariable analysis, a higher continuous HFIAS score was independently associated with greater odds of thinness (aOR 1.056, 95% CI 1.009–1.104, *p* = 0.019).

**Conclusion:**

School‐going adolescents in peri‐urban South Punjab experienced a very high burden of severe household food insecurity alongside substantial thinness, stunting, and overweight/obesity. These findings support adolescent‐focused food security screening, school‐based nutrition interventions, and locally targeted policies aligned with Sustainable Development Goal 2.

## Graphical abstract

A cross‐sectional study of 384 school‐going adolescents in peri‐urban South Punjab, Pakistan, found a high prevalence of severe household food insecurity (95.1%), including thinness, stunting, and overweight/obesity. These findings underscore the need for school‐based nutrition interventions and policies supporting Sustainable Development Goal 2.

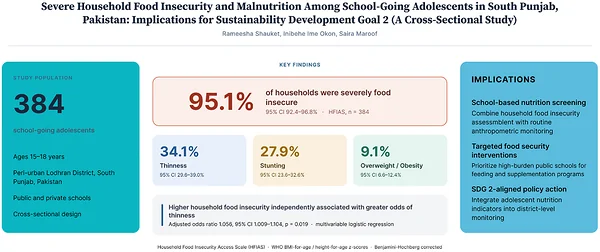

## 1 Introduction

Adolescence is a pivotal developmental stage marked by rapid physical growth, cognitive maturation, and psychosocial development. Adequate and sustained access to safe, diverse, and nutritious food during this period is essential for growth, school performance, health, and later‐life productivity. The Food and Agriculture Organization defines food security as a situation in which all people, at all times, have physical, social, and economic access to sufficient, safe, and nutritious food that meets dietary needs and food preferences for an active and healthy life [1]. Despite global commitments to end hunger, acute food insecurity remains a major global challenge [2].

Pakistan continues to face persistent food insecurity and malnutrition, driven by socioeconomic inequality, regional disparities, inflationary pressure, and structural vulnerabilities in food systems [3]. National and global indicators consistently show that food insecurity disproportionately affects rural and peri‐urban communities, where household income, food affordability, parental education, and livelihood instability influence access to adequate food [1, 4, 5]. Adolescents constitute a large and important segment of Pakistan's population, yet they are often less visible than children under 5 years and pregnant women in nutrition programming and research [6].

Existing Pakistani evidence suggests that adolescent food insecurity is an important but incompletely characterized public health issue: Previous studies have reported food insecurity and dietary diversity challenges among adolescents in rural Sindh and urban slum settings [4, 7], but evidence remains limited for school‐going adolescents in peri‐urban South Punjab, particularly regarding the simultaneous description of household food insecurity, BMI‐for‐age‐defined thinness and overweight/obesity, height‐for‐age‐defined stunting, and socioeconomic correlates. Recent studies from Karachi and Lahore similarly note that adolescent nutrition research remains concentrated in large cities and largely overlooks peri‐urban, school‐going populations, underscoring the need for more geographically representative, context‐specific evidence [8, 9].

The Sustainable Development Goals (SDGs), particularly SDG 2.1 and SDG 2.2, emphasize ending hunger and all forms of malnutrition by 2030 [10]; generating context‐specific evidence among adolescents is therefore necessary to inform school health programs, district‐level nutrition planning, and social protection policies. This study is conceptually guided by the FAO's four‐pillar Global Strategic Framework for Food Security, which identifies availability, access, utilization, and stability as the core dimensions of food security [11], and by an adapted model in which household food insecurity (the access dimension) and sociodemographic determinants (age, sex, family size, parental education and occupation, and household income) jointly shape adolescent nutritional status. The household is treated as the proximate environment through which access‐related deprivation is experienced by its adolescent members, with sociodemographic factors as moderators rather than independent causes; this motivates our focus on the Household Food Insecurity Access Scale (HFIAS) access dimension as the exposure most relevant to the individual‐level outcomes examined. This study was designed to estimate the prevalence of household food insecurity and anthropometric malnutrition among school‐going adolescents in peri‐urban South Punjab and to examine bivariate associations between food insecurity indicators, sociodemographic characteristics, and nutritional outcomes.

### 1.1 Objectives

- To assess the prevalence of household food insecurity among school‐going adolescents in peri‐urban South Punjab.
- To assess adolescent nutritional status using WHO BMI‐for‐age (BAZ) and height‐for‐age *z*‐score (HAZ) classifications.
- To examine bivariate associations between household food insecurity indicators and malnutrition categories among adolescents.

## 2 Methods

### 2.1 Study Design, Setting, and Participants

A descriptive cross‐sectional study was conducted in peri‐urban schools of Lodhran district, South Punjab, Pakistan, from December 2024 to May 2025. The study population consisted of school‐going adolescents aged 15–18 years enrolled in selected public and private schools; students with complete questionnaire and anthropometric information were included in the final analysis. Lodhran district has an agrarian economy, an estimated population of approximately 1.93 million, a literacy rate of approximately 51.7%, and the lowest Human Development Index ranking among Punjab's districts, reflecting substantial socioeconomic vulnerability relevant to household food access [12]. Punjab districts are administratively organized into tehsils, which are subdivided into municipal committees/councils (urban local government bodies responsible for municipal services) and union councils (the lowest tier of local government); the schools sampled were located within Dunyapur tehsil and Jallah Arein union council, selected as described under Section 2.3 [13].

### 2.2 Sample Size

The base minimum sample size was calculated using Cochran's formula for a single proportion under simple random sampling, *n*_0_ = *Z* ^2^ *pq*/*e* ^2^, with *Z* = 1.96, *p* = 0.60 (assumed food insecurity prevalence [14]), *q* = 0.40, and *e* = 0.05, giving *n*_0_ ≈ 369.

Because students were sampled through schools rather than independently (see Section 2.3), this base figure was adjusted for clustering using Kish's design effect formula, DEFF = 1 + (*m* − 1)*ρ* [15]. A study‐specific intra‐cluster correlation coefficient (*ρ*) cannot be estimated from this dataset, as only one tehsil and one union council were sampled; a literature‐informed value of *ρ* = 0.02 is, therefore, assumed, consistent with published estimates for school‐clustered anthropometric and food‐insecurity outcomes (typically 0.01–0.05). With the seven schools included in the study as the clustering unit (average cluster size *m* = 384/7 ≈ 55), DEFF ≈ 2.08, giving a clustering‐adjusted sample size of *n*_1_ = *n*_0_ × DEFF ≈ 768.

This figure was further adjusted for nonresponse using *n*_target = *n*_1_/RR, with RR = 97.6% (the response rate observed at data collection: 400 of 410 consented adolescents). This gives a fully corrected target of *n*_target = 768/0.976 ≈ 787.

Parental consent was obtained for 410 adolescents above the uninflated base target (*n*_0_ = 369) but below the fully corrected target (*n*_target ≈ 787), a shortfall addressed under Section 4.1. Of these 410, complete data were collected for 400 (10 unavailable on the day of data collection); 6 questionnaires were excluded for incompleteness (394 remaining); and 10 further records were excluded for anthropometric *z*‐scores outside the biologically plausible range per WHO AnthroPlus conventions, giving a final analytic sample of 384 (Figure 1): 369 → 787 (corrected target) → 410 (consented) → 400 (data collected) → 394 (complete) → 384 (final analytic sample).

**FIGURE 1:**
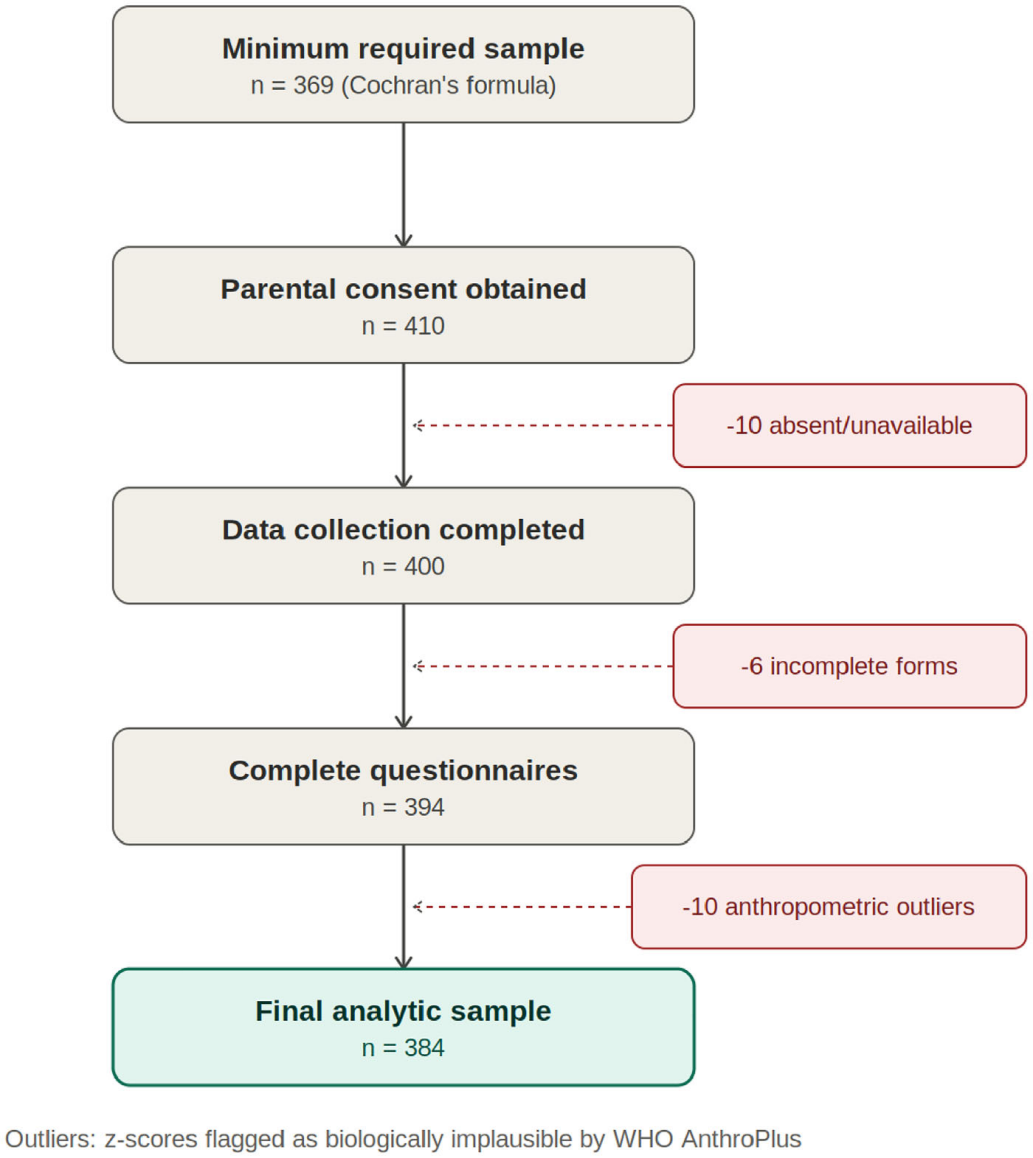
Participant flow from calculated minimum sample size to final analytic sample, showing sequential exclusions for nonresponse, incomplete forms, and biologically implausible anthropometric *z*‐scores.

### 2.3 Sampling Procedure

A multistage sampling approach was used. In the first stage, one of the three tehsils (municipal committees) comprising Lodhran district (Lodhran, Kahror Pakka, and Dunyapur) was selected by simple random sampling; Dunyapur was selected. In the second stage, one union council was selected by lottery method from the 22 union councils of Dunyapur tehsil; Jallah Arein union council was selected. In the third stage, all seven public and private schools located within Jallah Arein union council were included in the study (a census of schools within the selected union council, rather than a further sampling stage). In the final stage, students were selected from school enrollment (roll‐call) lists obtained from school principals/teachers by simple random sampling, stratified by school type. The final sample included 214 students (55.7%) from public schools and 170 (44.3%) from private schools. This design combines random selection of administrative units with simple random sampling of students within them and is best described as multistage cluster‐random sampling rather than a fully randomized, district‐wide probability sample, reflecting the study's logistical constraints.

### 2.4 Data Collection and Study Variables

Data were collected using a pretested, interviewer‐administered questionnaire. Interviewer administration was chosen over self‐administration because it improves completion rates and standardizes item interpretation across respondents of variable literacy, at some potential cost of social‐desirability bias [16]; trained data collectors read each item verbatim without prompting, and the questionnaire was pretested for clarity and cultural appropriateness among adolescents in a non‐study school before finalization. The questionnaire captured sociodemographic characteristics (age, gender, school grade, school type, family size, number of siblings, house ownership, and house construction), parental occupation and education, and monthly household income. Socioeconomic status was classified using the modified Kuppuswamy scale [17]. This scale derives a composite score from parental education, occupation, and income, yielding five categories from upper to lower, with higher scores indicating greater socioeconomic advantage [17]. The 2023 revision of the scale [17] scores monthly family income using seven nonsequential income bands (assigned scores of 1, 2, 3, 4, 6, 10, or 12; scores 5, 7, 8, 9, and 11 do not exist in the scale itself). Consequently, the income brackets reported in Table 1 do not run as a continuous sequence, and the apparent gaps between adjacent brackets reflect this scoring structure rather than missing or excluded data.

**TABLE 1:** Baseline sociodemographic, socioeconomic, and anthropometric characteristics (*N* = 384).

Characteristic	Category	Value
Age (years)	15	92 (24.0%)
	16	93 (24.2%)
	17	85 (22.1%)
	18	114 (29.7%)
Gender	Male	177 (46.1%)
	Female	207 (53.9%)
Number of family members	1–3	7 (1.8%)
	4–6	159 (41.4%)
	7–10	188 (49.0%)
	More than 10	30 (7.8%)
Number of siblings	1–3	88 (22.9%)
	4–6	231 (60.2%)
	7–10	60 (15.6%)
	More than 10	5 (1.3%)
School grade (class)	9th	135 (35.2%)
	10th	99 (25.8%)
	11th	98 (25.5%)
	12th	52 (13.5%)
School type	Public	214 (55.7%)
	Private	170 (44.3%)
Type of house	Own	370 (96.4%)
	Rent	14 (3.6%)
House construction	Concrete	287 (74.7%)
	Mud	97 (25.3%)
Father/Head‐of‐household occupation	Unemployed	37 (9.6%)
	Elementary occupation	130 (33.9%)
	Plant/Machine operator	12 (3.1%)
	Craft and related worker	5 (1.3%)
	Skilled agriculture worker	133 (34.6%)
	Skilled worker and shop	43 (11.2%)
	Clerk and accountant	3 (0.8%)
	Associate professional	4 (1.0%)
	Professional	17 (4.4%)
Mother's occupation	Unemployed	379 (98.7%)
	Elementary occupation	3 (0.8%)
	Plant/Machine operator	1 (0.3%)
	Craft and related worker	1 (0.3%)
Father/Head‐of‐household education	Illiterate	54 (14.1%)
	Primary education	68 (17.7%)
	Elementary education	119 (31.0%)
	High school education	92 (24.0%)
	Higher secondary education	35 (9.1%)
	Graduation	16 (4.2%)
Mother's education	Illiterate	147 (38.3%)
	Primary education	101 (26.3%)
	Elementary education	70 (18.2%)
	High school education	46 (12.0%)
	Higher secondary education	10 (2.6%)
	Graduation	10 (2.6%)
Family monthly income (PKR)^a^	≤23,800	70 (18.2%)
	23,804–71,298	117 (30.5%)
	71,301–118,844	86 (22.4%)
	118,847–148,329	75 (19.5%)
	148,332–177,814	20 (5.2%)
	237,695–356,537	7 (1.8%)
	>475,378	9 (2.3%)
Kuppuswamy socioeconomic class^b^	Upper class	3 (0.8%)
	Upper middle class	33 (8.6%)
	Lower middle class	111 (28.9%)
	Lower upper class	217 (56.5%)
	Lower class	20 (5.2%)
Height (cm), mean	Overall	161
Weight (kg), mean	Overall	47
HAZ, mean (median)	Overall	−0.99 (−0.79)
BAZ, mean (median)	Overall	−1.25 (−1.41)
HFIAS score, mean (median)^c^	Overall	15 (16)

Abbreviations: BAZ, BMI‐for‐age *z*‐score; HAZ, height‐for‐age *z*‐score; HFIAS, Household Food Insecurity Access Scale.

^a^Family monthly income was scored using the seven nonsequential income bands of the modified Kuppuswamy scale (2023 revision) [17]; scores 5, 7, 8, 9, and 11 do not exist in the scale, which accounts for the apparent gaps between adjacent brackets.

^b^Kuppuswamy socioeconomic class is the composite classification derived from parental education, occupation, and the income band above.

^c^Median reported alongside mean given the HFIAS score's skewed distribution (Shapiro–Wilk *p* < 0.001); see Section 2.

Household food access during the preceding 30 days was assessed using the HFIAS, a validated nine‐item instrument [18]. A household was operationally defined as the adolescent participant and the family members with whom they reside and share meals on at least 4 days per week; the adolescent served as respondent on behalf of the household, consistent with standard HFIAS guidance to select a single knowledgeable respondent. The HFIAS provided both individual occurrence‐domain information and overall household food insecurity prevalence categories (food secure, mildly, moderately, and severely food insecure). HFIAS reflects household‐level food access, whereas anthropometric outcomes reflect adolescent‐level nutritional status.

Anthropometric measurements were obtained using standard procedures. Height and weight were measured by trained data collectors using a calibrated stadiometer and digital scale following WHO protocols, with a subset of measurements independently repeated to check interobserver consistency. Nutritional status was calculated using WHO AnthroPlus software and classified using WHO growth reference standards for adolescents aged 5–19 years [19, 20, 21, 22]. BAZ were used to classify severe thinness (<−3), moderate thinness (−3 to −2), normal BMI‐for‐age (−2 to +1), overweight (>+1 to +2), and obesity (>+2), consistent with the WHO 2007 growth reference for ages 5–19 years, in which +1 SD and +2 SD correspond approximately to adult BMI thresholds of 25 and 30 kg/m^2^ at age 19. HAZ were used to classify severe stunting (<−3), moderate stunting (−3 to −2), normal height‐for‐age (−2 to +2), and tallness (>+2). The WHO 5–19‐year reference does not define severity gradients above +2 SD as it does for stunting; tallness is, therefore reported, here as a single descriptive category (>+2 SD) rather than a pathological classification. Thinness/severe thinness was considered acute malnutrition, stunting/severe stunting chronic malnutrition, and overweight/obesity overnutrition.

### 2.5 Statistical Analysis

Categorical variables were summarized using frequencies and percentages, and continuous variables using mean, median, mode, skewness, and kurtosis as appropriate. Chi‐square tests examined bivariate associations between sociodemographic characteristics, malnutrition categories, and HFIAS prevalence categories, and between individual HFIAS occurrence domains and malnutrition categories; a two‐sided *p* < 0.05 was considered significant. Fisher's exact test was used where expected cell counts were below five. Multivariable logistic regression, adjusted for age, sex, school type, family size, socioeconomic score, and school grade, tested the continuous HFIAS score against malnutrition outcomes. Because 91 bivariate tests were conducted across Figure 3, Benjamini–Hochberg correction was applied jointly across all *p* values to control the false discovery rate at *q* < 0.05, and only associations surviving correction were treated as robust. Chi‐square testing was used because primary variables were categorical; these bivariate analyses are exploratory, do not establish causality, and do not adjust for confounding, a limitation addressed in part by the multivariable analysis above. Multivariable analyses used binary logistic regression with a logit link (binomial family), fitted separately for each malnutrition outcome (Figure 4); the exposure of interest was the continuous HFIAS score, and outcomes were the three binary malnutrition indicators, and age, sex, school type, family size, Kuppuswamy socioeconomic score, and school grade were included as control variables to reduce confounding, with no a priori directional hypothesis specified for the latter.

## 3 Results

A total of 384 school‐going adolescents aged 15–18 years were included in the final analysis.

### 3.1 Participant Characteristics

Table 1 presents the baseline demographic characteristics. The mean age was 16.6 ± 1.1 years. The largest age group was 18 years (114/384, 29.7%), followed by 16 years (93/384, 24.2%), 15 years (92/384, 24.0%), and 17 years (85/384, 22.1%). Females constituted 207 participants (53.9%), whereas males constituted 177 (46.1%). Most participants belonged to large households: 188 (49.0%) reported 7–10 family members, and 231 (60.2%) reported 4–6 siblings. Public‐school students accounted for 214 participants (55.7%), and private‐school students for 170 (44.3%). Most adolescents lived in owned houses (96.4%) and concrete houses (74.7%).

### 3.2 Parental and Socioeconomic Characteristics

Table 1 also summarizes parental occupation, parental education, and household income. Fathers were most commonly skilled agricultural workers (133/384, 34.6%) or employed in elementary occupations (130/384, 33.9%), whereas nearly all mothers were not formally employed outside the home (379/384, 98.7%). Educational attainment was low, particularly among mothers: 147 (38.3%) were illiterate compared with 54 fathers (14.1%). Most households reported monthly income within lower income bands, with 117 (30.5%) reporting PKR 23,804–71,298 and 86 (22.4%) reporting PKR 71,301–118,844.

Table 1 also shows the Kuppuswamy socioeconomic categories. The most frequent category was lower upper class (217/384, 56.5%), followed by lower middle class (111/384, 28.9%); only 3 participants (0.8%) belonged to the upper class.

### 3.3 Continuous Variables

Table 1 also reports central tendency for key continuous variables. The overall mean height was 161 cm, and mean weight was 47 kg. The overall mean HAZ was −0.99, and mean BAZ was −1.25, indicating that the sample distribution was shifted below the WHO reference population. The overall mean HFIAS score was 15, and the median score was 16, supporting a high burden of household food insecurity.

### 3.4 Nutritional Status

Figure 2B presents malnutrition categories. On the basis of BAZ, 51 adolescents (13.3%) had severe thinness, and 80 (20.8%) had moderate thinness, giving a combined thinness/acute malnutrition prevalence of 131 adolescents (34.1%; 95% CI 29.6%–39.0%). Most adolescents had normal BMI‐for‐age (218/384, 56.8%). Overweight was observed in 27 adolescents (7.0%) and obesity in 8 (2.1%), giving a combined overweight/obesity prevalence of 35 adolescents (9.1%; 95% CI 6.6%–12.4%). On the basis of HAZ, 49 adolescents (12.8%) had severe stunting, and 58 (15.1%) had moderate stunting, yielding a combined prevalence of stunting/chronic malnutrition of 107 adolescents (27.9%; 95% CI 23.6%–32.6%). (95% Wilson confidence intervals reported above.)

**FIGURE 2:**
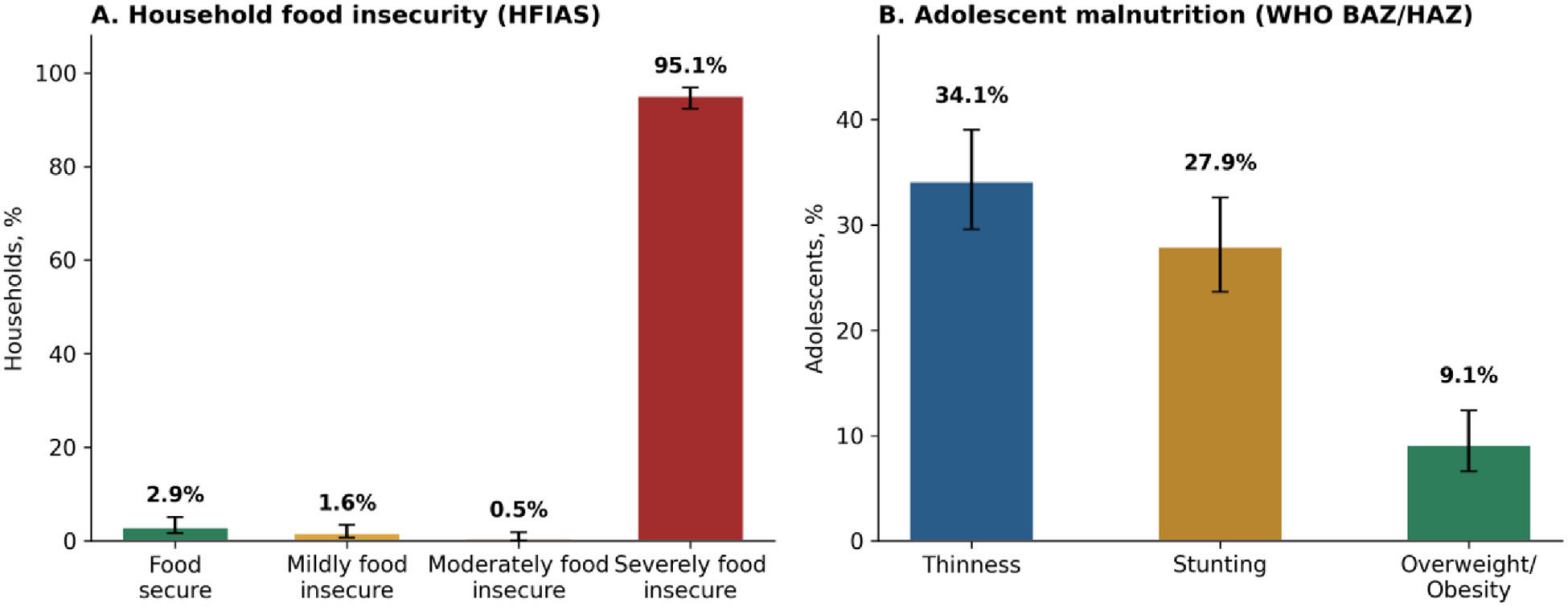
Prevalence of (A) household food insecurity (HFIAS) and (B) adolescent malnutrition (WHO BMI‐for‐age/height‐for‐age *z*‐scores), with 95% Wilson confidence intervals (*n* = 384).

### 3.5 Household Food Insecurity

Figure 2A summarizes HFIAS prevalence categories. More than 85% of adolescents reported household experiences corresponding to each HFIAS occurrence domain. The most frequently reported experiences were eating a smaller meal (357/384, 93.0%), eating foods they did not want to eat (356/384, 92.7%), eating fewer meals in a day (354/384, 91.1%), and having no food of any kind in the household (350/384, 91.1%). According to HFIAS prevalence categorization, 365 households (95.1%, 95% CI 92.4%–96.8%) were severely food insecure, 6 (1.6%, 95% CI 0.7%–3.4%) were mildly food insecure, 2 (0.5%, 95% CI 0.1%–1.9%) were moderately food insecure, and 11 (2.9%, 95% CI 1.6%–5.1%) were food secure.

### 3.6 Associations With HFIAS Prevalence Categories

Figure 3A presents bivariate associations between baseline characteristics, malnutrition categories, and HFIAS prevalence categories. Severe household food insecurity was significantly associated with age (*p* = 0.028), gender (*p* = 0.045), school grade/class (*p* < 0.001), school type (*p* = 0.037), head‐of‐household education (*p* = 0.007), family income (*p* = 0.014), and maternal education (*p* = 0.018). Moderate food insecurity was significantly associated with number of family members (*p* = 0.008) and family income (*p* < 0.001). Overall HFIAS prevalence categories were not significantly associated with stunting, thinness, or overweight/obesity in the bivariate analyses. After Benjamini–Hochberg correction across all 91 tests underlying Figure 3 (Table 2), only the association of school grade with food security status remained statistically robust. The nominally significant association between family income and moderate food insecurity (based on a chi‐square test) was re‐examined using Fisher's exact test, appropriate given that this comparison rested on a category of only two households; the Fisher's exact *p* value (0.019) did not survive Benjamini–Hochberg correction and is therefore not included among the robust findings. The remaining associations listed above did not survive correction and should be regarded as exploratory.

**FIGURE 3:**
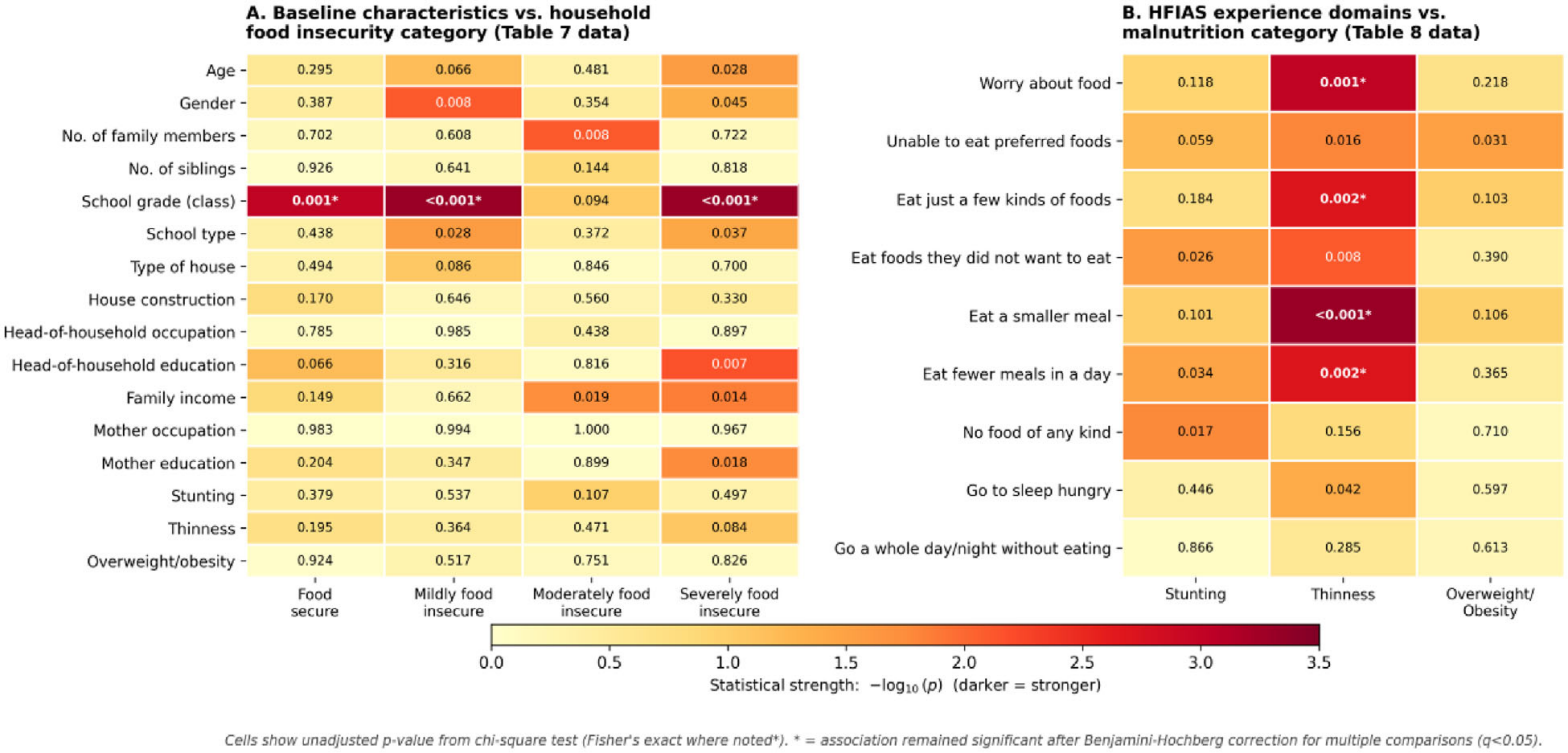
Heatmap of bivariate associations between (A) baseline characteristics and household food insecurity category, and (B) HFIAS experience domains and malnutrition category. Cell values are unadjusted *p* values (Fisher's exact where noted); darker shading indicates stronger statistical association (−log10 *p*). Asterisks (*) mark associations remaining significant after Benjamini–Hochberg correction for multiple comparisons (*q* < 0.05); see Table 2. HFIAS, Household Food Insecurity Access Scale.

**TABLE 2:** Bivariate associations remaining statistically significant after Benjamini–Hochberg false discovery rate correction across all 91 tests underlying Figure 3 (*q* < 0.05).

Source	Variable	Category/Outcome	*χ* ^2^	*p* (unadjusted)	*q* (FDR)
Figure 3A	School grade (class)	Food secure	16.13	0.001	0.018
Figure 3A	School grade (class)	Mildly food insecure	17.79	<0.001	0.015
Figure 3A	School grade (class)	Severely food insecure	30.84	<0.001	0.015
Figure 3B	Worry about food	Thinness	15.67	0.001	0.018
Figure 3B	Eat just a few kinds of foods	Thinness	14.39	0.002	0.026
Figure 3B	Eat a smaller meal	Thinness	18.12	<0.001	0.015
Figure 3B	Eat fewer meals in a day	Thinness	14.65	0.002	0.026

*Note:* An association between family income and moderate food insecurity was nominally significant by chi‐square (based on a category of only two households) but is not included here; retested using Fisher's exact test as appropriate for this sparse cell, it did not survive correction.

### 3.7 Associations Between Individual Food Insecurity Experiences and Malnutrition

Figure 3B presents associations between individual HFIAS occurrence domains and malnutrition categories. Stunting/chronic malnutrition was significantly associated with eating foods the household did not want to eat (*p* = 0.026), eating fewer meals in a day (*p* = 0.034), and having no food of any kind in the household (*p* = 0.017). Thinness/acute malnutrition was significantly associated with worry about food (*p* = 0.001), inability to eat preferred foods (*p* = 0.016), eating only a few kinds of foods (*p* = 0.002), eating foods the household did not want to eat (*p* = 0.008), eating a smaller meal (*p* < 0.001), eating fewer meals in a day (*p* = 0.002), and going to sleep hungry (*p* = 0.042). Overweight/obesity was significantly associated only with inability to eat preferred foods (*p* = 0.031). After correction (Table 2), only four domain‐level associations with thinness remained robust: worry about food, eating only a few kinds of foods, eating a smaller meal, and eating fewer meals in a day; no domain‐level association with stunting or overweight/obesity survived correction.

### 3.8 Multivariable Analysis

Because the HFIAS score and anthropometric indices departed significantly from normality (Shapiro–Wilk *p* < 0.001), Spearman correlation and Mann–Whitney *U* tests were used alongside multivariable logistic regression (Figure 4). The continuous HFIAS score was weakly but significantly correlated with HAZ (*ρ* = 0.147, *p* = 0.004) and was significantly associated with thinness status on the Mann–Whitney test but was not significantly correlated with BAZ overall or with overweight/obesity status. In multivariable logistic regression adjusting for age, sex, school type, family size, socioeconomic score, and school grade (variance inflation factors <2.0, indicating no problematic collinearity), a higher HFIAS score remained independently associated with greater odds of thinness (aOR 1.056, 95% CI 1.009–1.104, *p* = 0.019). Associations of HFIAS score with lower odds of stunting and overweight/obesity after adjustment ran counter to the a priori hypothesis and are interpreted with caution rather than as evidence of a protective effect. Private‐school attendance was independently associated with substantially higher odds of stunting and overweight/obesity and lower odds of thinness after adjustment.

**FIGURE 4:**
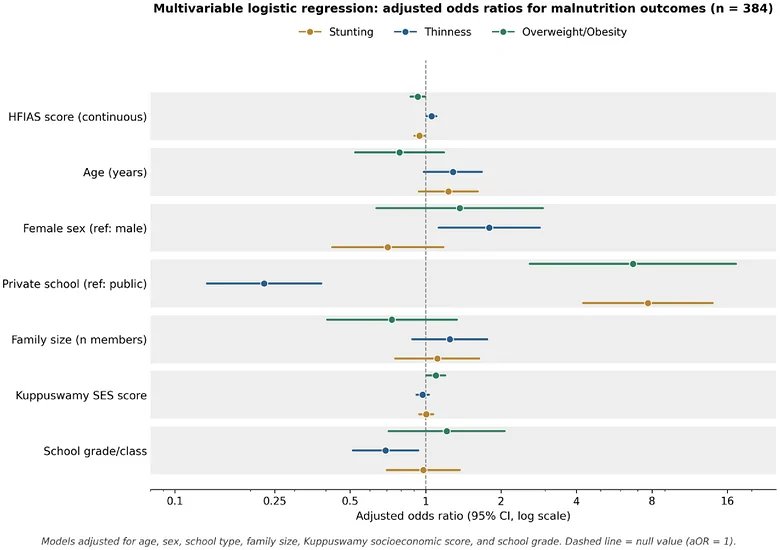
Forest plot of adjusted odds ratios (aOR, 95% CI) from multivariable logistic regression for stunting, thinness, and overweight/obesity, adjusted for age, sex, school type, family size, Kuppuswamy socioeconomic score, and school grade (*n* = 384). The exposure of interest was the continuous HFIAS score; control variables were included to reduce confounding, with no a priori directional hypothesis. Spearman correlation and Mann–Whitney *U* test results for the HFIAS score against anthropometric indices are reported in Section 3. HFIAS, Household Food Insecurity Access Scale.

## 4 Discussion

This study demonstrates a very high burden of severe household food insecurity among school‐going adolescents in peri‐urban South Punjab, Pakistan, alongside substantial anthropometric malnutrition. Nearly all households were classified as severely food insecure, approximately one‐third of adolescents had thinness, more than one‐quarter were stunted, and a smaller but important proportion had overweight or obesity. These findings are relevant to SDG 2 as contextual motivation for adolescent nutrition policy, although this study did not directly measure official SDG 2 monitoring indicators or assess progress toward specific targets.

The prevalence of stunting in this study was 27.9%, including moderate stunting in 15.1% and severe stunting in 12.8% of adolescents. Although lower than the stunting prevalence reported among adolescents in government schools in Sindh (47.4%) [7], it remains concerning because adolescence is a period when catch‐up growth may still be possible. Thinness/severe thinness was observed in 34.1% of participants, exceeding several reported adolescent undernutrition estimates and underscoring the nutritional vulnerability of the study population [7, 23].

Overweight or obesity was observed in 9.1% of adolescents. The coexistence of thinness, stunting, and overweight/obesity at the population level suggests a complex nutritional profile in which undernutrition remains dominant but overnutrition is also emerging; because this study did not assess micronutrient deficiencies or individual‐level double‐burden phenotypes, findings should be interpreted as population‐level coexistence of different malnutrition categories rather than direct evidence of a formal double burden at the individual level.

The HFIAS results indicate an exceptionally high level of household food insecurity, exceeding estimates reported in many other settings, including adolescent cohorts and household‐based studies from Pakistan and other low‐ and middle‐income contexts [7, 24, 25, 26]. This may reflect local economic vulnerability, food price pressures, household size, parental education, and livelihood patterns in the study area; because this was a school‐based sample from peri‐urban Lodhran, findings should be interpreted within this local context rather than generalized nationally. Reconstructing the HFIAS prevalence categories directly from item‐level responses using the standard scoring algorithm [18] reproduced the reported severe‐insecurity prevalence exactly and the remaining categories closely, confirming that the extreme skew reflects a correct application of the validated algorithm to the responses collected rather than a coding artifact, although reference‐period or social‐desirability effects in item responses cannot be excluded.

Although overall HFIAS prevalence categories were not significantly associated with stunting, thinness, or overweight/obesity, several individual HFIAS domains were nominally associated with malnutrition outcomes, likely reflecting limited variability in the overall categories: 95.1% of households were severely food insecure, leaving very small comparison groups and reduced power to detect differences across categories. Fisher's exact test confirmed the absence of association between severe food insecurity and each malnutrition outcome (stunting *p* = 0.607; thinness *p* = 0.134; overweight/obesity *p* = 0.688), corroborating rather than resolving the chi‐square result. However, treating the HFIAS score as continuous rather than categorical, multivariable analysis found a higher score independently associated with greater odds of thinness (Figure 4), suggesting the categorical null result partly reflects loss of information when a skewed continuous score is collapsed into four unevenly populated categories, rather than a true absence of association.

Bivariate associations between severe food insecurity and age, gender, school grade, school type, parental education, and family income are broadly consistent with prior literature linking socioeconomic vulnerability to household food access [5, 7]. However, only the association with school grade survived correction for multiple comparisons (Table 2) and should be regarded as the most credible of this group. The remaining associations did not survive correction and are exploratory, hypothesis‐generating observations warranting confirmation in adequately powered, correction‐robust designs. Lower parental education and household income may limit food purchasing power, dietary diversity, and access to health and nutrition information.

Public‐school students made up the majority of the sample (55.7%). Although the bivariate association between school type and severe food insecurity did not survive correction for multiple comparisons, multivariable logistic regression, which is not subject to the same multiple‐testing burden, independently found private‐school attendance strongly associated with all three malnutrition outcomes after adjustment (Figure 4); this adjusted analysis, rather than the unadjusted chi‐square test, should be regarded as the more credible evidence for a school‐type effect. This pattern is broadly consistent with other Pakistani evidence that undernutrition is more common among children and adolescents attending government/public schools, whereas overnutrition is more frequently documented among private‐school students from relatively advantaged households [8]; school type may, therefore, serve as a practical, low‐cost proxy for targeting school‐based nutrition screening toward public schools in similar peri‐urban settings, although the present data cannot fully disentangle school type from the underlying socioeconomic factors.

From a public health perspective, school‐based nutrition screening for adolescents should be strengthened to incorporate both household food insecurity assessment and anthropometric monitoring, with nutritionally vulnerable adolescents in high‐burden public schools prioritized for school feeding, nutrition supplementation, or linkage to social protection programs. Given Pakistan's commitments under SDG 2, adolescent nutrition indicators should be integrated more explicitly into district‐level SDG monitoring and school health programs. Longitudinal and mixed‐methods research is needed to clarify the temporal relationships between household food insecurity, dietary intake, and adolescent growth outcomes and to test whether school‐based interventions can reduce the burden documented here.

### 4.1 Limitations

This study has several limitations. First, the cross‐sectional design precludes causal inference between household food insecurity and nutritional outcomes. Second, HFIAS captures household food access over the preceding 30 days and does not directly measure individual dietary intake, nutrient intake, or food quality. Third, HFIAS responses were reported by the adolescent on behalf of the household, following standard HFIAS practice, whereas anthropometric, sociodemographic, and Kuppuswamy data were interviewer‐administered or directly measured, so only the HFIAS content is properly self‐reported. Because the same adolescent both reported household food access and underwent anthropometric assessment, exposure and outcome are linked through a common informant, partially offsetting the household‐individual conceptual gap discussed below; however, an adolescent respondent may have incomplete knowledge of household financial decisions compared with a parent, introducing its own source of measurement error. These responses may in any case be affected by recall or reporting bias. Fourth, micronutrient deficiencies, biochemical markers, and clinical signs of malnutrition were not assessed, so the study cannot address micronutrient‐related malnutrition or individual‐level double‐burden phenotypes. Pubertal stage, timing, and tempo, which can affect anthropometric *z*‐scores independent of nutritional status across this 15–18‐year age range, were also not assessed. Moreover, physical activity, infectious disease burden, or healthcare access, all of which could confound the food insecurity‐malnutrition relationship.

Fifth, given small expected cell counts in several comparisons, Fisher's exact test and multivariable logistic regression were added as supplementary analyses (Figure 4), and Benjamini–Hochberg correction was applied to the 91 bivariate tests underlying Figure 3, of which 7 associations remained significant (Table 2); the remaining nominally significant findings should be treated as exploratory. None of these analyses account for clustering of students within schools, which was also not incorporated into the original sample‐size calculation (see Section 2.2). Sixth, food insecurity was assessed at the household level via a single respondent, whereas nutritional status was assessed for the individual adolescent; because intrahousehold food allocation can vary by age, sex, and birth order, household‐level food insecurity does not necessarily equate uniformly into individual‐level nutritional deprivation. This conceptual mismatch should be borne in mind when interpreting these associations. Finally, the study was school‐based and conducted in one peri‐urban district; findings may not generalize to out‐of‐school adolescents, who are plausibly more vulnerable to food insecurity and malnutrition and are excluded from this sampling frame by design, or to other regions of Pakistan.

## 5 Conclusion

School‐going adolescents in peri‐urban Lodhran, South Punjab, experienced a very high prevalence of severe household food insecurity and a substantial burden of anthropometric malnutrition. Thinness, stunting, and overweight/obesity were all observed, indicating a complex nutritional profile at the population level. Overall HFIAS prevalence categories were not significantly associated with malnutrition outcomes, likely reflecting the extreme skew toward severe insecurity; however, select individual food‐insecurity experiences and the continuous HFIAS score were independently associated with thinness. These findings support the need for adolescent‐focused food security assessment, school‐based nutrition programs, and locally targeted interventions aligned with Sustainable Development Goal 2.

## Author Contributions

Conceptualization, study design, data collection, formal analysis, writing – original draft preparation, writing – review and editing, supervision, and final approval of the manuscript were performed by **Rameesha Shauket**, **Inibehe Ime Okon**, and **Saira Maroof**.

## Funding

The authors have nothing to report.

## Ethics Statement

This study was conducted in accordance with the ethical principles outlined in the Declaration of Helsinki for research involving human participants. Ethical clearance was obtained from the Institutional Research Ethics Committee of the Armed Forces Post Graduate Medical Institute, National University of Medical Sciences, Pakistan. The Institutional Review Board Ethical Clearance number was Ref: 544‐AAA‐ERC‐AFPGMI. Necessary administrative permission was obtained from relevant educational authorities and participating schools before data collection. Confidentiality and anonymity were maintained by avoiding the collection of personal identifiers and by securely storing study data with access limited to the research team.

## Consent

Written informed consent was obtained from parents or guardians, and participation was voluntary.

## Conflicts of Interest

The authors declare no conflicts of interest.

## Data Availability

The data that support the findings of this study are available from the corresponding author upon reasonable request.

## References

[1] FAO , Thre State of Food Security and Nutrition in the World 2024 Executive Summary (FAO, 2024), 10.4060/cd1254en.

[2] FAO , Anticipatory Action: Annual Report 2023 (FAO, 2024).

[3] S. Hashmi , N. F. Safdar , S. Zaheer , and K. Shafique , “Association Between Dietary Diversity and Food Insecurity in Urban Households: A Cross‐Sectional Survey of Various Ethnic Populations of Karachi, Pakistan,” Risk Management and Healthcare Policy 14 (2021): 3025–3035, 10.2147/RMHP.S284513. PMC8294809 34305415

[4] S. Sheikh , R. Barolia , A. Habib , I. Azam , R. Qureshi , and R. Iqbal , “ *“If the Food Is Finished After My Brother Eats Then We (girls) Sleep Hungry*.” Food Insecurity and Dietary Diversity Among Slum‐Dwelling Adolescent Girls and Boys in Pakistan: A Mixed Methods Study,” Appetite 195 (2024): 107212, 10.1016/j.appet.2024.107212. 38242362

[5] T. Mahmood , R. Kumar , T. M. Ali , N. Naeem , and S. Pongpanich , “Determinants of the Food Insecurity at Household Level in Pakistan: A Multilevel Model Approach,” PLoS ONE 18, no. 10 (2023): e0291343. 10.1371/journal.pone.0291343 PMC10553256 37797055

[6] WFO , Pakistan (World Food Programme, 2024), https://www.wfp.org/countries/pakistan.

[7] S. Sheikh , R. Iqbal , R. Qureshi , I. Azam , and R. Barolia , “Adolescent Food Insecurity in Rural Sindh, Pakistan: A Cross‐Sectional Survey,” BMC Nutrition 6, no. 1 (2020): 17, 10.1186/s40795-020-00343-w. PMC7098077 32226631

[8] D. S. A. Khan , J. K. Das , S. Zareen , et al., “Nutritional Status and Dietary Intake of School‐Age Children and Early Adolescents: Systematic Review in a Developing Country and Lessons for the Global Perspective,” Frontiers in Nutrition 8 (2021): 739447, 10.3389/fnut.2021.739447. PMC8848764 35187014

[9] S. Zafar , B. T. Shaikh , Z. I. Malik , and A. M. R. Ahmad , “Determinants of School‐Going Adolescent Girls' Health and Nutritional Status in District Malir, Karachi: A Baseline Cross‐Sectional Study,” Frontiers in Nutrition 11 (2024): 1510183, 10.3389/fnut.2024.1510183. PMC11747709 39839292

[10] UNO , SDG Country Profiles (UNO, 2025), https://unstats.un.org/sdgs/dataportal/countryprofiles/PAK#goal‐2.

[11] S. Devereux , C. Béné , and J. Hoddinott , “Conceptualising COVID‐19's Impacts on Household Food Security,” Food Security 12, no. 4 (2020): 769–772, 10.1007/s12571-020-01085-0. PMC7358330 32837651

[12] PBS (Pakistan Bureau of Statistics, 2023), https://www.pbs.gov.pk/census‐2017‐district‐wise/results/073.

[13] Government of Punjab , The Punjab Local Government Act 2013 (as Amended) (Government of Punjab), https://lgcd.punjab.gov.pk/system/files/Punjab%20Local%20Government%20Act%202013%20final.doc_.pdf.

[14] Z. Kibria , M. N. Khan , S. Aleem , and Z. U. Haq , “Linkages Between Poverty and Food Insecurity in Pakistan: Evidence From Urban and Rural Households in Peshawar,” Pakistan Journal of Medical Sciences 39, no. 2 (2023): 479–484, 10.12669/pjms.39.2.7122. PMC10025694 36950433

[15] L. Kish , Survey Sampling (Wiley, 1995), https://www.wiley.com/en‐us/survey‐sampling‐p‐9780471109495.

[16] M. A. Puhan , A. Ahuja , M. L. Van Natta , L. E. Ackatz , and C. Meinert , Studies of Ocular Complications of AIDS Research Group , “Interviewer Versus Self‐Administered Health‐Related Quality of Life Questionnaires—Does It Matter?” Health and Quality of Life Outcomes [Electronic Resource] 9 (2011): 30, 10.1186/1477-7525-9-30. PMC3100232 21554737

[17] M. Radhakrishnan and S. B. Nagaraja , “Modified Kuppuswamy Socioeconomic Scale 2023: Stratification and Updates,” International Journal of Community Medicine And Public Health 10, no. 11 (2023): 4415–4418, 10.18203/2394-6040.ijcmph20233487.

[18] J. Coates , A. Swindale , and P. Bilinsky , Household Food Insecurity Access Scale (HFIAS) for Measurement of Food Access: Indicator Guide: Version 3: (576842013‐001) , 10.1037/e576842013-001.

[19] M. de Onis , A. W. Onyango , E. Borghi , A. Siyam , C. Nishida , and J. Siekmann , “Development of a WHO Growth Reference for School‐Aged Children and Adolescents,” Bulletin of the World Health Organization 85, no. 9 (2007): 660–667, 10.2471/BLT.07.043497. PMC2636412 18026621

[20] WHO , Growth Reference 5–19 Years—Height‐for‐Age (5–19 Years) , https://www.who.int/tools/growth‐reference‐data‐for‐5to19‐years/indicators/height‐for‐age.

[21] WHO , Growth Reference 5–19 Years—BMI‐for‐Age (5–19 Years) , https://www.who.int/tools/growth‐reference‐data‐for‐5to19‐years/indicators/bmi‐for‐age.

[22] WHO , Malnutrition (WHO, 2025), https://www.who.int/health‐topics/malnutrition.

[23] J. Parida , B. S. Bagepally , P. K. Patra , S. Pati , H. Kaur , and S. K. Acharya , “Prevalence and Associated Factors of Undernutrition Among Adolescents in India: A Systematic Review,” BMC Public Health [Electronic Resource] 25, no. 1 (2025): 819, 10.1186/s12889-025-22054-2. PMC11871687 40022033

[24] M. S. Nnyepi , M. Phegelo , T. G. Ramolefhe‐Mutumwa , and D. Mmopelwa , “Association Between Household Food Insecurity (Access) Scores, Poverty Indicators and Child Malnutrition in Botswana,” Ecology of Food and Nutrition 62, no. 3–4 (2023): 130–145, 10.1080/03670244.2023.2202392. 37055878

[25] S. T. Yasmin , S. T. Yasmin , S. Sultan , and S. A. Hong , “Prevalence of Adolescent‐Reported Food Insecurity and the Determinants Including Coping Strategies Living in Urban Slum Communities of Bangladesh During the Era of COVID‐19: A Cross‐Sectional Study,” BMC Public Health [Electronic Resource] 23, no. 1 (2023): 2046, 10.1186/s12889-023-16984-y. PMC10588258 37858099

[26] S. Carries , L. N. Sigwadhi , A. Moyo , C. Wagner , C. Mathews , and D. Govindasamy , “Associations Between Household Food Insecurity and Depressive Symptomology Among Adolescent Girls and Young Women During the COVID‐19 Pandemic in South Africa,” Adolescents 4, no. 1 (2024): 185–199, 10.3390/adolescents4010013.

